# Golgi-independent routes support protein disulfide isomerase externalization in vascular smooth muscle cells

**DOI:** 10.1016/j.redox.2017.04.034

**Published:** 2017-05-03

**Authors:** Thaís L.S. Araujo, Carolina G. Fernandes, Francisco R.M. Laurindo

**Affiliations:** From the Vascular Biology Laboratory, Heart Institute (InCor), University of São Paulo School of Medicine, Postal code: 05403-000, São Paulo, Brazil

**Keywords:** csPDI, cell-surface PDI, BFA, brefeldin-A, CytD, cytochalasin-D, Mon, monensin, Methy, methylamine, pecPDI, peri/epicellular PDI, TgPDI, transgenic mice overexpressing PDIA1, TNFα, tumor necrosis factor-α, VSMC, vascular smooth muscle cell, Protein disulfide isomerase, Vascular smooth muscle cell, Unconventional protein traffic, Endoplasmic reticulum, Cell-surface chaperones

## Abstract

Extracellular pools of intracellular molecular chaperones are increasingly evident. The peri/epicellular(pec) pool of the endoplasmic reticulum redox chaperone protein disulfide isomerase-A1(PDI) is involved in thrombosis and vascular remodeling, while PDI externalization routes remain elusive. In endothelial cells, vesicular-type PDI secretion involves classical and unconventional pathways, while in platelets PDI exocytosis involves actin cytoskeleton. However, little is known about pecPDI in vascular smooth muscle cells(VSMC). Here, we showed that VSMC display a robust cell-surface(cs) PDI pool, which binds to cs independently of electrostatic forces. However, contrarily to other cells, soluble secreted PDI pool was undetectable in VSMC. Calcium ionophore A23187 and TNFα enhanced VSMC csPDI. Furthermore, VSMC PDI externalization occurred via Golgi-bypass unconventional route, which was independent of cytoskeleton or lysosomes. Secreted PDI was absent in ex vivo wild-type mice aortas but markedly enhanced in PDI-overexpressing mice. Such characterization of VSMC pecPDI reinforces cell-type and context specific routes of PDI externalization.

## 1 Introduction

Molecular chaperones from the cytosol or endoplasmic reticulum (ER) are known to be externalizated in many cell types, such as immune cells [1], [2], tumor cells [3], hepatocytes, pancreatic cells, platelets and endothelial cells (EC) [4]. Protein disulfide isomerase (PDIA1 or PDI) is a major ER redox chaperone responsible for thiol oxidoreductase and isomerase effects in its nascent protein clients [5]. The peri/epicellular PDI (=pecPDI) pool exerts many extracellular functions involving thiol redox and possibly chaperone effects [6], [7]. Some pecPDI-associated functions include: a) disulfide bond reduction of HIV glycoprotein gp120, tumor endothelial marker-5, αvβ3 integrins, tissue factor, αMβ2 and β1 integrin [6]; b) dithiol oxidation of alpha5 integrin [8] and c) thiol-disulfide isomerization of ADAM17 and αIIbβ3 [6]. Such effects associate with pecPDI-mediated regulation of viral infection [9], [10], platelet activation [11], [12], thrombosis [7], [13], [14] and expansive vascular remodeling [15]. PecPDI inhibition is increasingly investigated as a novel promising anti-thrombotic strategy [16], through cell-impermeable compounds that target PDI b'x or b' substrate-binding domains [17], [18], while targetting of the redox-active a' domain also has an attractive anti-thrombotic potential [19], [20]. Such pecPDI implications enhanced the interest to understand PDI externalization routes across distinct cell types, which remain elusive so far. In platelets, PDI secretion occurs through an exocytosis process dependent on cytoskeletal actin [21]. In EC, PDI is detectable in intracellular vesicles [22], suggesting a similar process. We showed recently, through an extensive pharmacological screening, that PDI subcellular traffic in EC involves both classical and unconventional routes that are not actin cytoskeleton-dependent and disclosed some differences in the mode of externalization for cell-surface vs. secreted PDI pools [8]. However, the precise molecular pathways involved in these processes are unclear. Moreover, little is known about pecPDI in vascular smooth muscle cells (VSMC). We previously reported that pecPDI exerts an anti-constrictive vascular remodeling effect during post-injury vessel repair in association with extracellular matrix and cytoskeleton reorganization in EC and VSMC, together with β1integrin thiol reduction [15]. Also, pecPDI neutralization in VSMC stimulates procoagulant microparticle release in P2rx7^–/–^ mice [23]. Moreover, VSMC may be a source of extracellular PDI also through active or passive cell death-related processes in vascular disease [13], [14]. However, a clear characterization of the extracellular PDI pool, as well as the mode of PDI externalization in VSMC, remains poorly understood. Here, we investigated the pattern of pecPDI externalization and major types of secretory pathways involved in this process.

## 2 Material and methods

### 2.1 Cell culture

Rabbit aortic VSMC from a previously established selection-immortalized line were maintained in growth medium (DMEM, with 10% fetal bovine serum) at 37 °C in 5% CO_2_ atmosphere [24]. Primary VSMC from rabbit aorta were isolated and maintained as described [15].

### 2.2 Classical secretory and cystoskeleton pathway disruption

All treatments were performed in serum-free DMEM with each concentration indicated in the respective legend. Validated concentrations of each agent were used as guides for our selection, adjusting according to cell viability and, in some cases, associated cellular changes (e.g., cytoskeletal effects with cytochalasin and cell vesiculation effect with methylamine).

### 2.3 Cell-surface biotinylation

VSMC were seeded at 3.5x10^5^cells in 60 mm petri dishes in a 3 mL DMEM containing 10%serum fetal bovine for 24 h. Treatments, as indicated in each legend, were performed in serum-free medium. Biotinylation experiments with EZ-Link sulfo-NHS(N-hydroxysuccinimido)-biotin (Thermo-Scientific) for 1 h at 4 °C were performed as described [25]. The reaction was stopped with 50 mM Tris/HCl pH=7.5 for 10 min at 4 °C. After cell lysis (50 mM Tris-HCl, 1% Triton X-100, 150 mM NaCl) for 1 h at 4 °C, cells were incubated overnight with streptavidin magnetic beads (Promega), followed by pull- down, washing with PBS to remove proteins that were not biotinylated, incubation in sample buffer for 30 min, boiling for 5 min and then SDS-PAGE, followed by western blot. All western blots were performed with anti-PDIA1 RL90 from Thermo-Scientific.

### 2.4 Cytotoxicity assay : annexin-V/propidium iodide

VSMC were seeded at 6x10^4^cells in 12-well plates for 24 h, followed by drug treatments for the same time used in confocal assays. After trypsin-harvesting, cells were washed with PBS, incubated with annexin-V 1:20 (Invitrogen) for 15 min at 25 °C, followed by propidium iodide (0.1 mg/mL), before reading. The annexin-V and propidium iodide stainings were evaluated by flow cytometry.

### 2.5 Confocal immunofluorescence

VSMC were seeded (1.5x10^4^/well) onto glass coverslips in 24-well plates for 24 h. After treatments, cells were fixed in 4% paraformaldehyde (20 min at 25 °C), rinsed in PBS, permeabilized or not in 0.1% Nonidet p40 (30 min at 37 °C) and blocked with 2% BSA in PBS for 30 min at 37 °C. Primary antibodies were incubated overnight at 4 °C and secondary antibodies, Alexa Fluor 488 (1:200) and Alexa 546 (1:200), for 2 h at 25 °C, diluted in PBS containing 1% BSA. Working dilutions of primary antibodies were as follows: anti-PDIA1 (SPA891; 1:200/ Enzo), anti-Erp72 (1:200/Abcam) and Phalloidin (1:100/Invitrogen). Nuclei were stained with DAPI (Invitrogen, 10 µg/mL) or Hoechst-33258 (1:200). Immunostained coverslips were mounted on microscope slides using glycerol/PBS (1:2,v/v). Cells were visualized through an inverted laser confocal microscope (Zeiss LSM510-Meta) from our local “Rede Premium” Facility. Pinhole was adjusted according to objective and sample thickness. Co-localization images represent the sum of 5–7 slices (0.5-µm thickness) for each coverslip. Images were processed using LSM Image Browser or Fiji [4] software. Co-localization analysis was estimated by calculating Pearson’s correlation coefficient between the indicated image channels, selecting full cell images and using area co-localization in the LSM 510 Expert Mode software. Pearson’s correlation coefficient was calculated for each experiment as the mean of five images for each condition. All images shown are representative from at least three independent experiments.

### 2.6 Conditioned medium from aorta in organ culture

Aortas were collected from anesthesized 4–9 month-old mice that were either wild type or constitutively transgenic for ubiquitous PDI overexpression. Details of this model have been described elsewhere (Fernandes et al., manuscript under review). Briefly, PDIA1-overexpressing mice (from FVB background) present no obvious macroscopic phenotype and breed normally. Presence of the transgene was identified in each case by genotyping and an associated PDI Myc tag. Mice were euthanized in a CO_2_ chamber, and their abdominal aortas immediately removed and cut into fragments (2–3 mg), followed by incubation in DMEM (70 μL) at 37 °C in 95% O_2_/5% CO_2_ atmosphere in 0.6 mL eppendorfs. After 4 h, the aortas were removed, the conditioned medium centrifuged at 3000*g* for 10 min at 4 °C to remove debris/cells and the supernatant kept at −80 °C until use for ≤3 weeks. The aliquot of conditioned medium was normalized according to weight, so that a medium amount equivalent to 2 mg tissue was incubated in Laemmli buffer for 30 min before boiling at 100 °C for 5 min, followed by western blot. Western analysis was performed with anti-PDI RL90, Hsp70 (Abcam, 3A3), myc-tag (Cell Signalling, 71D10) or β-actin (sigma).

### 2.7 Statistical analysis

Data are mean±SEM. Densitometric analysis of all immunoblots was performed using Odyssey Li-Cor Software. Each experiment was independently repeated at least three times. Data were analyzed by paired *t*-test to compare two groups or one-way ANOVA plus Student-Newman-Keuls multiple-range test, to compare ≥3 groups. A p-value <0.05 was considered significant. All analyses were performed using GraphPad Prism v.5.

## 3 Results

### 3.1 VSMC exhibit a cell-surface, but not a secreted PDI pool in basal conditions

**Fig. 1:**
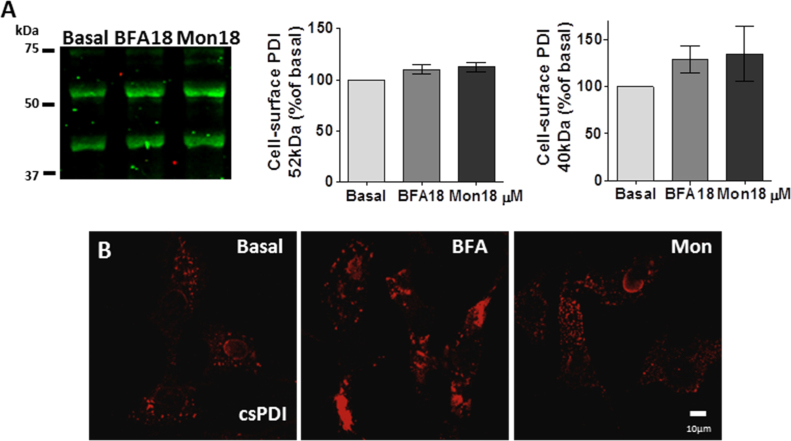
**Cell-surface PDI is not externalized by the ER-Golgi pathway in VSMC.** (A) Primary VSMC were treated for 4.5 h with BFA 18 µM or Mon 18 µM and underwent cell-surface biotinylation, followed by staining with anti-PDI RL90. All data are mean±SEM from three separate experiments. (B) Immunofluorescence staining of PDI (in red) in non-permeabilized VSMC in basal condition or after treatment with BFA 10 µM or Mon 10 µM for 4.5 h. All immunofluorescence data were obtained with anti-PDI SPA891, which recognizes the C-terminal domain. All images are representative of at least three independent experiments. (For interpretation of the references to color in this figure legend, the reader is referred to the web version of this article.)

We first characterized the extracellular PDI pools present in VSMC. Importantly, in clear contrast to EC cultivated under the same conditions [8], we were unable to detect soluble secreted PDI in VSMC conditioned medium even after a prolonged 24-h incubation. Meanwhile, csPDI was detectable through biotinylation experiments in primary VSMC (Fig. 1A, Basal) and by confocal microscopy in non-permeabilized VSMC from a cell line (Fig. 1B, Basal). Of note, we detected a strong additional csPDI band of ca.40 kDa, consistent with partial lysis, despite the use of protease inhibitors. Such band is completely absent in EC analyzed under similar conditions [8]. This biotinylation assay was previously validated in our laboratory in endothelial cells, in which we showed cell-surface thrombomodulin expression [8].

### 3.2 The classical secretory pathway does not seem the predominant route of PDI externalization in VSMC

**Fig. 2:**
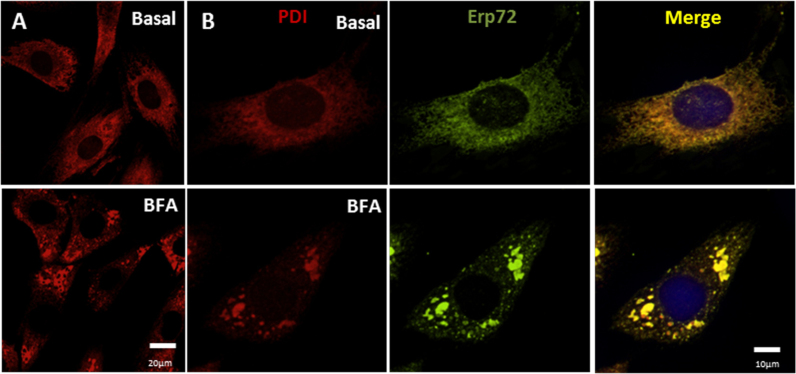
**BFA promotes plaque/conglomerate-like PDI pattern in VSMC**. (A) Immunofluorescence staining of PDIA1 (red) and Erp72 (green) in permeabilized VSMC in basal condition or after treatment with BFA 10 µM for 4.5 h. Data were performed as independent triplicates. (B) Similar experiment (A) with P0 image obtained from summatory image gallery to depict stained proteins in all plans, allowing to calculate Pearson correlation coefficient as described in Material and Methods. All images shown are representative of at least three independent experiments. (For interpretation of the references to color in this figure legend, the reader is referred to the web version of this article.)

Next, we investigated the role of classical ER-Golgi pathway in csPDI externalization in VSMC. For that, we used blockers of the classical secretory pathway: Brefeldin A (BFA) or Monensin (Mon), which have been well-validated in the literature for this purpose [26], including experiments in VSMC [27]. BFA blocks retrograde transport from Golgi to ER, leading to prominent Golgi disruption [26], while Mon increases Golgi pH, affecting Golgi-dependent traffic [28], [29]. Importantly, our results showed that BFA (18 µM, 4.5 h) and Mon (18 µM, 4.5 h) did not decrease csPDI levels, assessed through biotinylation experiments (Fig. 1A) and confocal microscopy in non-permeabilized cells (Fig. 1B). Intriguingly, BFA promoted a reorganization of csPDI from a normal punctate staining to a plaque/conglomerate-like pattern (Fig. 1B). These results were also reproduced in primary VSMC. Of note, such BFA-induced effect in PDI redistribution was also observed at the intracellular level, visualized after cell permeabilization, although less prominently (Fig. 2A). However, this BFA-associated PDI redistribution pattern was cell-type specific, being absent in EC [8] or in mouse embryonic fibroblasts [29]. Meanwhile, a similar pattern was also observed for another PDI family member, namely Erp72 (Fig. 2B) and Pearson correlation coefficient analysis revealed strong colocalization between csPDI and csErp72 at baseline (0.68±0.09) and after BFA incubation (0.59±0.09, mean±SEM). Importantly, cell death did not account for csPDI presence since annexin-V and propidium iodide cellular stainings were similar vs. non-treated cells at baseline (11.8±0.89%) or after BFA incubation (14.5±0.49, mean±SEM).

### 3.3 Actin cystoskeleton or lysosomal integrity are not required for PDI externalization in VSMC

**Fig. 3:**
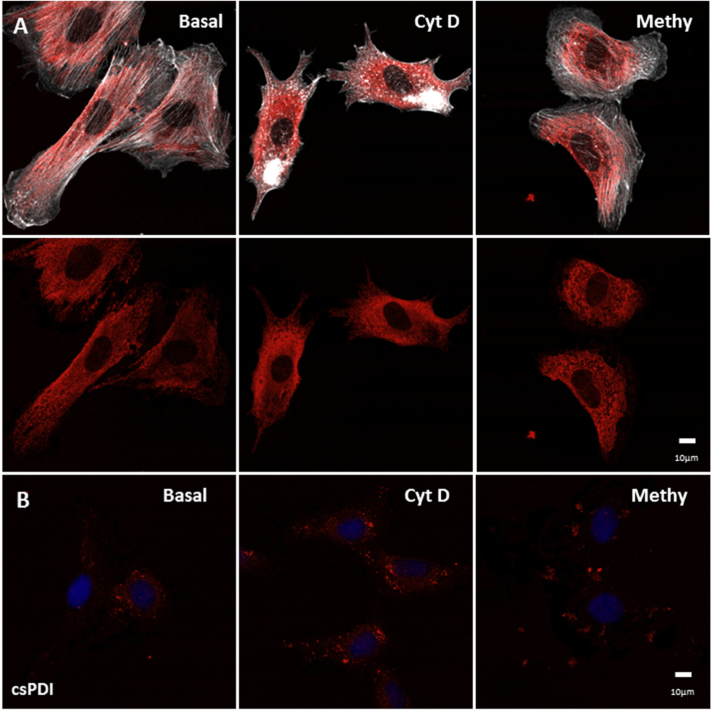
**VSMC PDI externalization was unaltered by cytoskeletal disorganization or lysosome impairment.** (A) Immunofluorescence staining of PDI (red) and phalloidin, showing F-actin (white) in permeabilized VSMC at baseline or after incubation with CytD 0.5 μM and Methylamine 10 mM for 1.5 h. (B) Immunofluorescence staining of PDI (red) in non-permeabilized VSMC in basal condition or after treatment as in (A). All images shown are representative of at least three independent experiments. (For interpretation of the references to color in this figure legend, the reader is referred to the web version of this article.)

Given the role of actin cytoskeleton in PDI externalization in platelets [21], we investigated whether cytoskeleton disruption could affect PDI externalization in VSMC. Cytochalasin D (CytD) promoted disruption of actin stress fibers in VSMC (Fig. 3A, CytD), as expected [30] and consequently slightly altered PDI distribution with respect to the absence of PDI staining along F-actin stress fibers (Fig. 3A, CytD). Methylamine, which promotes neutralization of lysosomal compartment, [31] triggered vesiculation in VSMC, an effect associated with drastic alteration in PDI intracellular distribution pattern (Fig. 3A). However, neither disruption of actin cytoskeleton nor lysosomal integrity promoted any change in csPDI levels (Fig. 3B).

### 3.4 Calcium ionophore A2387 or TNFα stimulate PDI externalization in VSMC

**Fig. 4:**
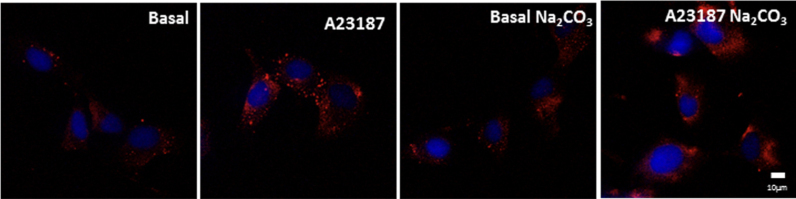
**Cell-surface PDI is increased by calcium ionophore A23187 in VSMC.** Immunofluorescence staining of PDI (red) in non-permeabilized VSMC at baseline or after incubation with calcium ionophore A23187 5 μM for 0.5 h. When indicated, VSMC were incubated with sodium carbonate 0.1 M, pH=9.0, for 10 min, followed by immunofluorescence staining of PDIA1 (red) in non-permeabilized VSMC. All images shown are representative of at least three independent experiments. (For interpretation of the references to color in this figure legend, the reader is referred to the web version of this article.)

**Fig. 5:**
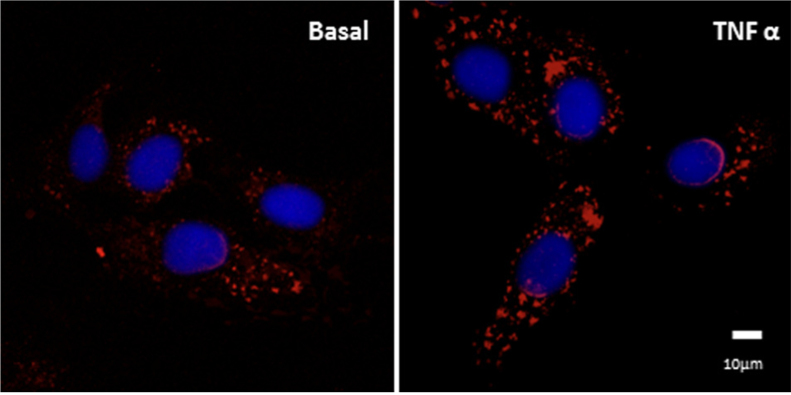
**VSMC cell-surface PDI is enhanced by TNF-α.** Immunofluorescence staining of PDI (red) in non-permeabilized VSMC at baseline or after incubation with TNF-α 50 ng/mL for 0.5 h. All images shown are representative of at least three independent experiments. (For interpretation of the references to color in this figure legend, the reader is referred to the web version of this article.)

PDI secretion is stimulated by calcium ionophore A23187 in EC [8], [22], chinese hamster ovary cells [32], platelets [33] and fibroblasts [34]. Our results (Fig. 4) indicate that A23187 enhances csPDI levels in VSMC in a way independent of cell damage, as assessed through annexin-V and propidium iodide staining (Baseline, 11.8±0.9% vs A23187, 15.0±1.7, p=NS). To investigate whether electrostatic interactions could support csPDI attachment to the cell surface, we tested the effects of sodium carbonate washing, which, however, did not change basal or A23187-stimulated csPDI levels (Fig. 4). As the role of proinflammatory cytokines in PDI externalization in VSMC is unknown, we examined the effects of tumor necrosis factor α (TNFα) incubation on csPDI levels. TNFα incubation (50 ng/mL, 0.5 h) promoted PDI externalization in VSMC (Fig. 5).

### 3.5 PDI overexpression promotes soluble PDI secretion in mice aorta *ex vivo*

**Fig. 6:**
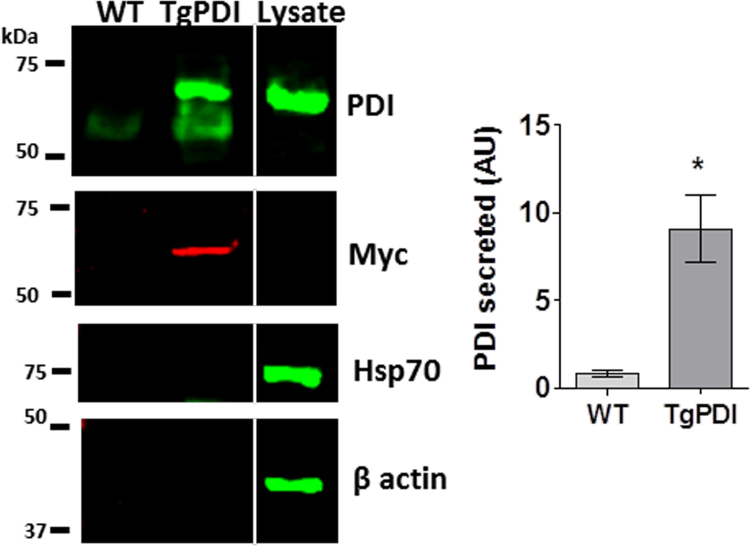
**PDI secretion from ex vivo aorta segments from wild-type or PDI-overexpressing mice.** Conditioned medium was prepared as described in Material and Methods, followed by western blot assay. The same membrane was stained with anti-PDI RL90, anti-Hsp70, anti-Myc tag and β actin. Data are mean±SEM from four animals, * p<0.05, *t*-test.

To gain further insight into the physiological pattern of PDI externalization by VSMC, we turned to a model of short-term *ex vivo* organ culture. We had previously shown that csPDI is detectable in rabbit aorta segments in this condition [15]. However, given the lack of detectable soluble PDI secretion in cultured VSMC (Fig. 1), we furthered our investigation into whether PDI would be secreted from aortic tissue. Although one cannot exclude that EC and some remaining adventitial cells may contribute to such PDI secretion, VSMC make up the vast majority of cells in these aortic segments. Our results indicate that abdominal aorta from wild type (WT) mice secreted an essentially undetectable amount of soluble PDI in basal culture conditions (Fig. 6). As a control, we injured the aorta with tweezers and readily detected soluble PDI in conditioned medium of wild-type aorta (not shown). However, aortic segments collected from transgenic mice with ubiquitous constitutive PDI overexpression (TgPDI, see methods) secreted a significant amount of soluble PDI, in a way unassociated to cell damage, given the absence of Hsp70 and β-actin staining in the conditioned medium (Fig. 6). Similar results were obtained using segments from crossa/thoracic aorta.

## 4 Discussion

Extracellular PDI is revealing important roles in vascular-related pathophysiological conditions such as platelet activation [12], thrombosis [16], inflammation [35] and vascular remodeling after injury [15]. Vascular-related physiological roles of pecPDI are less explored, though we described that pecPDI mediates alpha5 integrin oxidation in EC submitted to physiological levels of laminar arterial shear stress [8]. VSMC may be a possible source of pecPDI during thrombus formation [13], [14] and likely play a major role in pecPDI-mediated effects in vascular remodeling [15]. Also, VSMC are the source of matrix vesicles displaying PDI expression upon mineralization stimuli [36]. However, a more clear characterization of VSMC-associated PDI externalization remained an open issue. Our work contributes to support the existence of a robust csPDI pool in VSMC. It is well known from other cell types that PDI is externalized despite keeping the C-terminal KDEL ER-retrieval sequence. VSMC surface-associated PDI (Fig. 1) also contained the KDEL retention sequence, since the antibody used for this assay was targeted to the C-terminal domain of PDI. Of note, the majority of PDI interaction with the cell surface did not involve electrostatic forces (Fig. 4), similar to what we observed for EC csPDI, though not for EC Hsp70 (unpublished results from our group). In all such cases, however, the major mechanisms accounting for PDI cell-surface retention likely involve its interaction with other proteins, e.g., integrins [37]. The small amounts of csPDI observed in VSMC are in line with those observed for EC [8]. Although detection of csPDI in EC depicts only full-length csPDI at 55 kDa [8], VSMC csPDI reproducibly depict a strong band of ca. 40 kDa, while we detected a similar band when analyzing csPDI in iliac arteries [15]. Together, we believe this shorter band represents a cell-specific cleavage which is not artifactual.

An important feature of PDI externalization route in VSMC is its Golgi independence (Fig. 1), assessed through resistance to BFA and Mon incubation. Interestingly, in EC csPDI pool externalization was partially decreased by BFA and Mon [8], revealing cell type-specific differences in the mode of PDI externalization. Additionally, BFA-induced conglomerate pattern of csPDI appeared VSMC-specific, as it was not observed in EC [8]. The actin cystoskeleton is essential to platelet PDI externalization [21], however disruption of actin stress fibers increases PDI secretion in VSMC (Fig. 3B), similarly to EC [8]. Together, these new data add to previous literature information to suggest that despite cell-specific characteristics, unconventional pathways predominate regarding PDI externalization pathways. In all cases, direct or indirect evidence implicates exocytic pathways involving vesicular intermediates, which in both VSMC (Fig. 3) and EC [8] are likely distinct from acidic vesicles, since methylamine did not affect PDI externalization. Delivery of vesicles from cortical ER to the cell surface remains an open possibility for both cell types, which require further investigation. Whether VSMC csPDI sensitivity to calcium ionophore A23187 (Fig. 4), well-known for other cell types [22], [32], [34], [38], reflects calcium-triggered vesicle exocytosis also deserves more investigation. The observed increase in VSMC with TNFα (Fig. 5) might synergize with EC csPDI regarding neutrophil recruitament during vascular inflammation, given that TNFα is a pro-inflammatory cytokine known to increase ICAM-1 levels in EC [39] and integrin αMβ2 activity is positively regulated by pecPDI [40].

Another intriguing difference between VSMC and both EC and platelets is the absence of soluble-secreted PDI pool in VSMC, which was observed also in conditioned medium from aorta. Interestingly, PDI overexpression leads to secretion of endogenous as well as Myc tagged-PDI (Fig. 6) showing that PDI secretion may to some extent depend on overall intracellular PDI levels, although we cannot exclude that a specific subcellular compartment accounts for such process. While here PDI overexpression was forced, massive increases in PDI expression have been observed in vascular disease conditions [15]. It did not escape us that PDI overexpression could overload KDEL receptor [4] and allow enhanced PDI secretion. Overall, these data further reinforce previous suggestions in EC [8] that secreted PDI may behave as a differentially-regulated pool vs. csPDI.

In conclusion, our data provide clear evidence for a robust csPDI pool in VSMC, which is supported via unconventional Golgi-bypass secretion route(s). In addition, secreted PDI pool appears negligible in baseline VSMC, contrarily to other cell types, although it may increase upon enhanced intracellular PDI expression. These data further implicate cell type and context-specific routes of PDI externalization and help advance into their yet elusive nature.
